# Impact Assessment of GNSS Spoofing Attacks on INS/GNSS Integrated Navigation System

**DOI:** 10.3390/s18051433

**Published:** 2018-05-04

**Authors:** Yang Liu, Sihai Li, Qiangwen Fu, Zhenbo Liu

**Affiliations:** School of Automation, Northwestern Polytechnical University, Xi’an 710072, China; lisihai@nwpu.edu.cn (S.L.); fuqiangwen@nwpu.edu.cn (Q.F.); zhnbliu@gmail.com (Z.L.)

**Keywords:** GNSS spoofing, impact assessment, integrated navigation system

## Abstract

In the face of emerging Global Navigation Satellite System (GNSS) spoofing attacks, there is a need to give a comprehensive analysis on how the inertial navigation system (INS)/GNSS integrated navigation system responds to different kinds of spoofing attacks. A better understanding of the integrated navigation system’s behavior with spoofed GNSS measurements gives us valuable clues to develop effective spoofing defenses. This paper focuses on an impact assessment of GNSS spoofing attacks on the integrated navigation system Kalman filter’s error covariance, innovation sequence and inertial sensor bias estimation. A simple and straightforward measurement-level trajectory spoofing simulation framework is presented, serving as the basis for an impact assessment of both unsynchronized and synchronized spoofing attacks. Recommendations are given for spoofing detection and mitigation based on our findings in the impact assessment process.

## 1. Introduction

Global Navigation Satellite System (GNSS) spoofing is a technique to trick the victim receiver into generating an erroneous position fix and/or clock offset, by deliberately broadcasting legitimate-appearing false satellite signals [1,2]. Civil GNSS service is vulnerable to spoofing due to the open structure and low power of GNSS satellite signals [1]. GNSS spoofing is difficult to detect and may result in more serious situations than jamming, since the user may not be aware of it [3].

GNSS spoofing is not a new topic. When Global Positioning System (GPS) was first declared full operational in 1995, researchers from MITRE examined GPS spoofing and its countermeasures [4]. The Volpe report, released by US Department of Transportation clearly pointed out the potential for spoofing attacks [1]. However, the GNSS community gave little attention to this threat, until a portable GPS spoofer was successfully developed and demonstrated [5]. Several proof-of-concept spoofing tests have been carried out against unmanned aerial vehicles [6,7], super yachts [8], cars and smartphones [6,9]. Besides these filed demonstrations, the Iran-US RQ-170 incident and the recently reported GPS problem in central Moscow [10] and the Black Sea [11], have further intensified the interest in this area. With open source GPS signal simulators available online and the fast developing software-defined radio technology, GNSS spoofing has become “*not only feasible but also affordable*” [12].

In response to the emerging GNSS spoofing threats, many contributions have been made, which can be divided into spoofing implementation, assessment, detection and mitigation. (1) *Spoofing implementation* focuses on the mechanisms of spoofing attacks, serving as the basis for impact analysis and validation of anti-spoofing methods. Several research groups have implemented the so-called receiver-spoofer as defined in [5], while the others rely on simulators/repeaters for spoofing simulation. (2) *Spoofing impact assessment* evaluates the spoofing effects on GNSS receivers and systems that depend on, or relate to, GNSS. (3) Research on *spoofing detection and mitigation*, which take up the majority of the GNSS community’s efforts has resulted in the development of methods to revel, classify and eliminate spoofing threats. These methods can be classified into signal-processing based, encryption-based, drift-monitoring-based, signal geometry-based and multipronged spoofing defense strategies, as summarized in [2]. Detailed reviews of spoofing detection and mitigation methods can also be found in [13,14,15,16].

Among the existing spoofing defenses, inertial measurement unit (IMU) or inertial navigation system (INS) aided methods have been developed. With specific force and angular rate being the only required measurements for dead-reckoning, INS is self-contained and thus, invulnerable to radio frequency interference, like jamming and spoofing. The widely used INS/GNSS integrated navigation system inherits this nature to some extent. Attitude solutions of INS have been used to detect GNSS spoofing with a dual antenna configuration [17]. INS/GNSS Kalman filter innovations have been investigated to detect spoofing attacks [18,19,20,21]. The popularity of IMU/INS makes these methods very promising as many vehicle systems (land/sea/air/space) rely on them to serve a broad variety of applications.

The 2001 Volpe report raised the issue that “little publicly available information or test results exist concerning the response of commercial receivers to spoofing” [1]. This belongs to the impact assessment and has been answered by many contributions over the last decade. The US Department of Homeland Security hosted the 2017 GPS Equipment Testing for Critical Infrastructure, which provided manufacturers of commercial GPS receivers with a valuable testing opportunity against live-sky spoofing. Although the GNSS community has begun to carry out a standard and unified process to thoroughly assess the spoofing impacts on GNSS receivers, unfortunately, there is still a lack of comprehensive spoofing impact assessment on the widely used INS/GNSS integrated navigation system. When INS is integrated with GNSS, it can be affected by spoofing attacks due to cascade effects of the integration mechanism. To address the impact assessment problem for INS/GNSS systems under spoofing attacks, we focus on the response of the integrated navigation system’s Kalman filter to spoofed GNSS measurements, including its error covariance, innovation sequence and inertial sensor bias estimation. Understanding the behavior of INS/GNSS systems in the face of GNSS spoofing is crucial to hardening the integrated system against spoofing threats. In order to avoid the complexity and high cost of setting up live-sky GNSS spoofing tests (which must be authorized) for INS/GNSS systems, we propose a simple and straightforward high fidelity simulation framework. Based on the clues found in the impact assessment process, recommendations are given for potential spoofing detection and mitigation methods. In this paper, we make three main contributions:

- Based on the authentic and spoofed signal model, a comparison between unsynchronized and synchronized GNSS spoofing attacks is given. A framework for a measurement-level trajectory spoofing simulation is proposed, which simplifies the process of impact assessment for integrated navigation systems.
- We systematically analyze the impact of spoofing on the integrated navigation filter’s error covariance, innovation and inertial sensor bias estimation, revealing how the conventionally used Kalman filer responds to spoofing attacks.
- According to the impact assessment, we make recommendations for the cautious use of (1) error covariance for integrity monitoring, and (2) calibrated inertial sensors for pure INS solutions. Spoofing detection methods based on innovations and inertial sensor bias monitoring are suggested.

The remainder of this paper is organized as follows. Section 2 briefly introduces the INS/GNSS model used in this paper; Section 3 introduces basic GNSS spoofing attack modes and presents two methods for measurement-level spoofing simulation; Section 4 focuses on the impact assessment analysis and our recommendations for spoofing detection and mitigation; Section 5 verifies our findings through simulations, and the conclusions are given in Section 6.

## 2. INS/GNSS Integrated Navigation Model

In this paper, for simplicity, the loosely coupled INS/GNSS integrated navigation system is considered. As we focused on the Kalman filter’s error covariance matrix, innovation and inertial sensor bias estimation, the analytical derivation and analysis in the following sections are also applicable to the tightly coupled system. The underlying INS/GNSS integrated system is a nonlinear time-varying system, so the extended Kalman filter was used for the integration. The error state, instead of the total state, of the navigation system was chosen as the state vector, which is defined as [22]

(1) $$x\left(t\right)={\left[\begin{array}{ccccc} \phi^{T} & {\left(\delta v^{n}\right)}^{T} & {\left(\delta p\right)}^{T} & {\left(\varepsilon^{b}\right)}^{T} & {\left(\nabla^{b}\right)}^{T} \end{array}\right]}^{T},$$

where $\phi ={\left[\phi_{E},\phi_{N},\phi_{U}\right]}^{T}$ is the misalignment angle vector; $\delta v^{n}={\left[\delta v_{E}^{n},\delta v_{N}^{n},\delta v_{U}^{n}\right]}^{T}$ is the velocity error vector; $\delta p={\left[\delta L,\delta \lambda ,\delta H\right]}^{T}$ is the position error vector, consisting of latitude, longitude and height components; and $\varepsilon^{b}={\left[\varepsilon_{R}^{b},\varepsilon_{F}^{b},\varepsilon_{U}^{b}\right]}^{T}$ and $\nabla^{b}={\left[\nabla_{R}^{b},\nabla_{F}^{b},\nabla_{U}^{b}\right]}^{T}$ represent the gyro and accelerometer bias vectors, respectively. Note that the subscripts *E*, *N*, *U* represent the east, north and up components in the navigation frame, respectively. The subscripts, *R*, *F*, *U* represent the right, forward and up components in the body frame, respectively.

After the linearization, the system dynamic model and measurement model can be written as [22]

(2) $$\left\{ \begin{array}{l} \dot{x}\left(t\right)=F\left(t\right)x\left(t\right)+w\left(t\right)\\ z\left(t\right)={\tilde{p}}_{\mathrm{INS}}-{\tilde{p}}_{\mathrm{GNSS}}=H\left(t\right)x\left(t\right)+v\left(t\right) \end{array}\right.,$$

where F(t) is the system matrix; w(t) is the process noise vector; z(t) represents the difference between the position solution of INS ${\tilde{p}}_{\mathrm{INS}}$ and GNSS ${\tilde{p}}_{\mathrm{GNSS}}$; H(t) is the measurement matrix; and v(t) represents the noise of GNSS solutions. The system matrix, F(t), takes the form [23]

(3) $$F\left(t\right)=\left[\begin{array}{ccccc} F_{11} & F_{12} & F_{13} & -C_{b}^{n} & 0_{3}\\ F_{21} & F_{22} & F_{23} & 0_{3} & C_{b}^{n}\\ 0_{3} & F_{32} & F_{33} & 0_{3} & 0_{3}\\ 0_{3} & 0_{3} & 0_{3} & 0_{3} & 0_{3}\\ 0_{3} & 0_{3} & 0_{3} & 0_{3} & 0_{3} \end{array}\right],$$

where $F_{ij}$ is a 3-by-3 matrix; $C_{b}^{n}$ is a 3-by-3 body frame to navigation frame rotation matrix; and $0_{3}$ is a 3-by-3 zero matrix. The details of $F_{ij}$ are given in Appendix A [23]. The measurement matrix is a constant matrix and is defined as $H\left(t\right)=\left[\begin{array}{ccccc} 0_{3} & 0_{3} & I_{3} & 0_{3} & 0_{3} \end{array}\right]$, in which $I_{3}$ is a 3-by-3 identity matrix.

## 3. GNSS Spoofing Attacks and Measurement-Level Simulation

### 3.1. GNSS Spoofing Attacks

**Figure 1:**
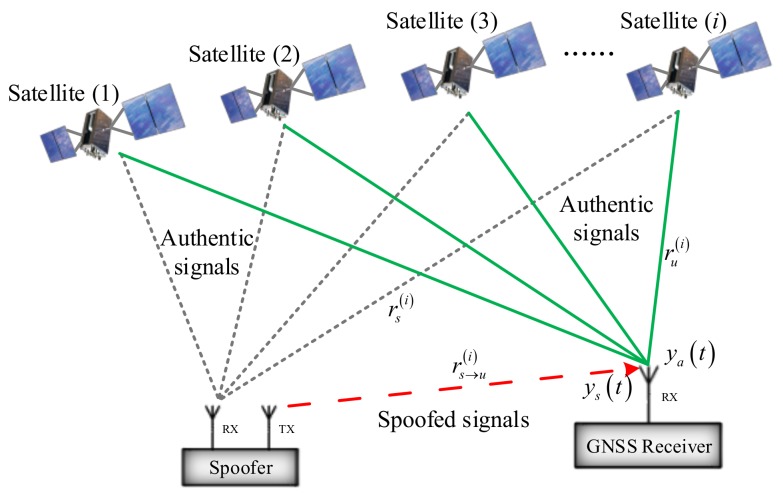
Global Navigation Satellite System (GNSS) spoofing attack illustration.

A brief illustration of a GNSS spoofing attack is given in Figure 1.

Assuming that the spoofing attack starts at $t_{\mathrm{Init}}$, the received GNSS signal, y(t), at the receiver can be expressed as [2],

(4) $$y\left(t\right)=y_{a}\left(t\right)+y_{s}\left(t\right)+n\left(t\right),\;t\geq t_{\mathrm{Init}},$$

where $y_{a}\left(t\right)$ and $y_{s}\left(t\right)$ are authentic and spoofed signals, respectively. n(t) is the receiver noise. Taking GPS L1 coarse/acquisition code spoofing as an example, the received authentic signal $y_{a}\left(t\right)$ is given by

(5) $$y_{a}(t)=\sum_{i}^{N_{a}}\sqrt{2P_{a}^{\left(i\right)}}D_{a}^{\left(i\right)}\left(t-\tau_{a}^{\left(i\right)}\right)C_{a}^{\left(i\right)}\left(t-\tau_{a}^{\left(i\right)}\right)\sin\left(2\pi \left(f_{1,a}^{\left(i\right)}+f_{d,a}^{\left(i\right)}\right)t+\theta_{1,a}^{\left(i\right)}\right).$$

The received spoofed signal $y_{s}\left(t\right)$ has a similar form:

(6) $$y_{s}(t)=\sum_{i}^{N_{s}}\sqrt{2P_{s}^{\left(i\right)}}D_{s}^{\left(i\right)}\left(t-\tau_{s}^{\left(i\right)}\right)C_{s}^{\left(i\right)}\left(t-\tau_{s}^{\left(i\right)}\right)\sin\left(2\pi \left(f_{1,s}^{\left(i\right)}+f_{d,s}^{\left(i\right)}\right)t+\theta_{1,s}^{\left(i\right)}\right),$$

where the superscript (*i*) represents the *i*th satellite, the subscripts *a* and *s* represent the authentic and spoofed signals, respectively. *N* is the number of visible satellites; *P* is the signal power; *D* is the navigation data bit stream; *C* is the pseudo random noise (PRN) code sequence; *τ* is the signal propagation delay; *f*_1_ is the GPS L1 signal frequency; *f*_d_ is the Doppler shift; and *θ*_1_ is the received initial carrier phase.

There are several essential features of GNSS spoofing attacks [2,24]. The spoofing signals must have exactly the same PRN code sequence and signal frequency to those of the authentic signals. The number of spoofed satellites is generally equal to the number of authentic signals; if not, the introduction of inconsistency may trigger the receiver autonomous integrity monitoring algorithm. The structure of the navigation data bit stream should be the same as that of the authentic stream, but the content may be manipulated to introduce the intended deception. The received initial carrier phase is generally unknown and it is practically impossible for the spoofer to align with the authentic, unless a very precise (cm-level) relative position between the spoofer and the target receiver is known. The Doppler shift of the spoofed signal is not necessarily the same as the authentic signal; however, it must be consistent with the spoofer’s own code phase variation.

GNSS spoofing attacks can be divided into unsynchronized and synchronized spoofing, based on whether the spoofed signals are time synchronized (code-phase aligned) with the authentic ones [16]. This classification highlights the error characteristics of spoofing attacks on raw GNSS measurements, which has an analogy to the step and ramp faults in integrity analysis. If the spoofer cannot know the target receiver’s position accurately (meter-level to maximum of a half code chip) [16], only unsynchronized spoofing attacks can be carried out. For synchronized spoofing attacks, the spoofer accurately knows the target receiver’s real-time position, which makes it possible for the spoofed signals’ delay and the Doppler shift to be consistent with the authentic signal at the target receiver end [25]. A comparison between these two spoofing attack modes is given in Appendix B. Understanding the characteristics of different spoofing attacks is essential to achieve high fidelity spoofing simulation for impact assessment and defense verification.

### 3.2. Measurement-Level Spoofing Attack Simulation

To analyze the signal-level response of the GNSS receivers to spoofing attacks, a sophisticated signal-level spoofing simulator is necessary. However, for an impact assessment on INS/GNSS integrated navigation systems focusing on the Kalman filter performance with spoofed GNSS measurements, a measurement-level simulator is sufficient. The measurement-level simulation of spoofing attacks has the advantage of easy and rapid implementation, avoiding the complexity and high cost of setting up live-sky GNSS spoofing tests which must be authorized. The measurement-level simulator generates raw GNSS observations based on given a constellation, error model and predefined trajectory profile (each epoch with a seven-dimensional noise free PVT solution). Using the synchronized spoofing attack as an example, we show how the pseudorange measurements of the target receiver are affected, which also shows how the spoofer or spoofing simulator construct pseudorange measurements for a synchronized spoofing attack. While for unsynchronized spoofing, the spoofing simulator can generate arbitrary measurements without considering the relationship with the authentic signals.

A raw pseudorange measurement for the *i*th satellite $\rho^{\left(i\right)}$ at time *t* is modeled as

(7) $$\rho^{\left(i\right)}=c\tau^{\left(i\right)}+c\left(\left(t+\delta t_{u}\right)-\left(t+\delta t^{\left(i\right)}\right)\right),$$

where $\tau^{\left(i\right)}$ is the signal transmission delay; *c* is the speed of light; and $\delta t_{u}$ and $\delta t^{\left(i\right)}$ are the receiver and satellite clock offset, respectively. For authentic signals, the signal delay, $\tau_{a}^{\left(i\right)}$, consists of the delay caused by the geometric range, $r_{u}^{\left(i\right)}$, the ionospheric effect, $I_{a}^{\left(i\right)}$, and the tropospheric, effect $T_{a}^{\left(i\right)}$, that is,

(8) $$\tau_{a}^{\left(i\right)}=\frac{r_{u}^{\left(i\right)}}{c}+I_{a}^{\left(i\right)}+T_{a}^{\left(i\right)}.$$

Considering the receiver noise, $n_{\rho ,a}^{\left(i\right)}$, the authentic pseudorange is obtained with

(9) $$\rho_{a}^{\left(i\right)}=r_{u}^{\left(i\right)}+c\left(\delta t_{u,a}-\delta t_{a}{}^{\left(i\right)}\right)+cI_{a}^{\left(i\right)}+cT_{a}^{\left(i\right)}+n_{\rho ,a}^{\left(i\right)}.$$

For spoofed signals, the signal delay, $\tau_{s}^{\left(i\right)}$, is

(10) $$\tau_{s}^{\left(i\right)}=\frac{r_{s}^{\left(i\right)}+r_{s\to u}}{c}+I_{s}^{\left(i\right)}+T_{s}^{\left(i\right)}+\nabla \tau_{proc}^{\left(i\right)}+\nabla \tau_{ctrl}^{\left(i\right)},$$

where $r_{s}^{\left(i\right)}$ is the geometric range from the spoofer to the satellite, and $r_{s\to u}$ is the geometric range from the spoofer to the target receiver (common for all satellites). $\nabla \tau_{proc}^{\left(i\right)}$ and $\nabla \tau_{ctrl}^{\left(i\right)}$ are the signal processing delay and controlled signal delay, respectively. Assuming a common atmospheric effect for the spoofer and target receiver, Equation (10) can be written as

(11) $$\begin{array}{ll} \tau_{s}^{\left(i\right)} & =\frac{r_{u}^{\left(i\right)}+\left(r_{s}^{\left(i\right)}+r_{s\to u}-r_{u}^{\left(i\right)}\right)}{c}+I_{a}^{\left(i\right)}+T_{a}^{\left(i\right)}+\nabla \tau_{proc}^{\left(i\right)}+\nabla \tau_{ctrl}^{\left(i\right)}\\ & =\underset{\tau_{a}^{\left(i\right)}}{\underset{︸}{\frac{r_{u}^{\left(i\right)}}{c}+I_{a}^{\left(i\right)}+T_{a}^{\left(i\right)}}}+\underset{\nabla \tau_{s}^{\left(i\right)}}{\underset{︸}{\frac{\left(r_{s}^{\left(i\right)}+r_{s\to u}-r_{u}^{\left(i\right)}\right)}{c}+\nabla \tau_{proc}^{\left(i\right)}+\nabla \tau_{ctrl}^{\left(i\right)}}} \end{array},$$

where $\nabla \tau_{s}^{\left(i\right)}$ is the additional signal delay introduced by the spoofer at the target receiver end. For a receiver/spoofer with the ability to predict the navigation bits, $\delta t_{adv\_ctrl}^{\left(i\right)}$ is introduced to represent the advanced prediction time by the spoofer to compensate for additional signal delays. Then, we get

(12) $$\begin{array}{ll} \rho_{s}^{\left(i\right)} & =c\left(\tau_{a}^{\left(i\right)}+\nabla \tau_{s}^{\left(i\right)}\right)+c\left(\left(t+\delta t_{u,a}\right)-\left(t+\delta t_{adv\_ctrl}^{\left(i\right)}+\delta t_{a}^{\left(i\right)}\right)\right)\\ & =c\tau_{a}^{\left(i\right)}+c\left(\delta t_{u,a}-\delta t_{a}{}^{\left(i\right)}\right)+c\nabla \tau_{s}^{\left(i\right)}-c\delta t_{adv\_ctrl}^{\left(i\right)}\\ & =\rho_{a}^{\left(i\right)}+c\nabla \tau_{s}^{\left(i\right)}-c\delta t_{adv\_ctrl}^{\left(i\right)} \end{array}.$$

Assuming that $\nabla \tau_{proc}^{\left(i\right)}$ is the same for all satellites, the superscript of $\nabla \tau_{proc}^{\left(i\right)}$ will thus be omitted thereafter. In $\nabla \tau_{s}^{\left(i\right)}$, both the transmission delay, $r_{s\to u}/c$, and the signal processing time, $\nabla \tau_{proc}$, will be estimated as the clock offset. Finally, we obtain

(13) $$\rho_{s}^{\left(i\right)}=\rho_{a}^{\left(i\right)}+\underset{c\delta {t^{\prime }}_{u,s}}{\underset{︸}{c\nabla \tau_{proc}^{}+r_{s\to u}}}+\underset{c{\tau^{\prime }}_{s}^{\left(i\right)}}{\underset{︸}{r_{s}^{\left(i\right)}-r_{u}^{\left(i\right)}+c\nabla \tau_{ctrl}^{\left(i\right)}-c\delta t_{adv\_ctrl}^{\left(i\right)}}},$$

where $\delta t_{u,s}^{\prime }$ and $\tau_{s}^{\prime }{}^{\left(i\right)}$ are the additional clock offset and signal delay introduced by the spoofing attacks. While $\nabla \tau_{proc}$ can be calibrated by the spoofer [26], $r_{s\to u}$, $r_{s}^{\left(i\right)}$ and $r_{u}^{\left(i\right)}$ should be calculated in real-time with the knowledge of the spoofer’s and target receiver’s locations. The spoofer adjusts $\nabla \tau_{ctrl}^{\left(i\right)}$ and $\delta t_{adv\_ctrl}^{\left(i\right)}$ to control the pseudorange measurements of the target receiver. The composite terms, $\delta t_{u,s}^{\prime }$ and $\tau_{s}^{\prime }{}^{\left(i\right)}$, are used to control the time and position solution of the target receiver, respectively.

For measurement-level spoofing simulation, we define the authentic and spoofed trajectory profiles as $tr_{a}\left(t\right)$ and $tr_{s}\left(t\right)$, respectively. The two trajectories satisfy

(14) $$tr_{s}\left(t\right)-tr_{a}\left(t\right)=\{\begin{array}{c} s\left(t\right),\;t\geq t_{\mathrm{Init}}\\ \;0,\;t<t_{\mathrm{Init}} \end{array},$$

where s(t) is the desired spoofing profile which can be further defined as s(t)=α(t)+b. The term α(t) can be any time-related function and b is a constant vector. With $\alpha \left(t_{\mathrm{Init}}\right)=0$ and b=0, synchronized spoofing attacks can be simulated. As a good start, α(t) can be a simple ramp function similar to integrity analysis in the GNSS community. For unsynchronized spoofing, the term b is used to represent the jumps introduced to the authentic GNSS solutions. There are two ways to achieve the measurement level spoofing attack simulation:

- When s(t) is determined, the components $\delta t_{u,s}^{\prime }$ and $\tau_{s}^{\prime }{}^{\left(i\right)}$ in Equation (13) can be calculated accordingly. They are added to the authentic measurements generated based on $tr_{a}\left(t\right)$ to construct the spoofed measurements. In this way, the critical parameters of the spoofer can also be simulated.
- If we only focus on the INS/GNSS integrated navigation system, a simpler method can be used without direct calculation of $\delta t_{u,s}^{\prime }$ and $\tau_{s}^{\prime }{}^{\left(i\right)}$. This is done by directly feeding $tr_{a}\left(t\right)$ and $tr_{s}\left(t\right)$ to the GNSS measurement-level simulator as two independent trajectories. A switch from $tr_{a}\left(t\right)$ to $tr_{s}\left(t\right)$ during the simulation can easily introduce the spoofed measurements to the integrated navigation system.

## 4. Impact Assessment and Recommendations

### 4.1. Error Covariance

The diagonal elements of the Kalman filter error covariance matrix, $P_{k}$, represent the error variance of each state estimation when the filter is properly modeled. They are often used to evaluate the performance of the integrated navigation system. In face of GNSS spoofing attacks, there is a necessity to analyze its impacts on the Kalman filter $P_{k}$ calculation.

**Figure 2:**
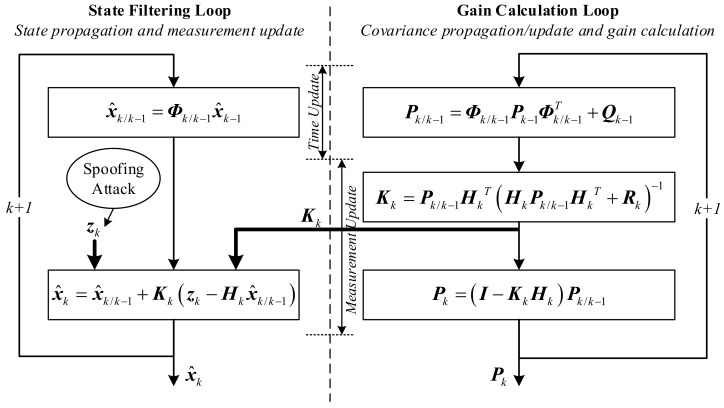
Standard Kalman filter implementation with spoofing attacks introduced.

A standard Kalman filter implementation is given in Figure 2 with spoofing attacks introduced. GNSS spoofing attacks are injected directly into the state filtering loop by adding a spoofing profile to $z_{k}$. For a typical spoofing attack, a positioning error of several tens of meters to several kilometers is sufficient to cause serious problem for safety critical applications. It should be noted that, in order to analyze the effects solely caused by the spoofing attacks, the system dynamic/measurement model (including the associated model parameters such as the process noise covariance matrix, $Q_{k}$ and the measurement noise covariance matrix, $R_{k}$) and the filter initialization process, are assumed to be exactly the same under spoofed and authentic conditions in the impact assessment analysis. This ensures that all the other factors that may influence the Kalman filter’s performance are excluded.

As shown in Figure 2, the only link between the state filtering loop (left) and the gain calculation loop (right) is the Kalman filter gain matrix, $K_{k}$. Intuitively, this is a one-way connection which means that the left loop is affected by the right but not vice versa. For linearized Kalman filter implementation, in which the system equation is linearized along the predefined reference trajectory, the gain calculation loop (which can be done off-line) is completely independent to the state filtering loop. For the more commonly used extended Kalman filter, there is a minor difference between the open-loop feedforward and closed-loop feedback mechanisms. If the feedforward integration is used, in which only the outputs of the integrated navigation system are corrected, the pure INS-based reference trajectory remains unaffected, and thus, the state filtering loop is also completely independent of the gain calculation loop. However, for the feedback integration, the error states estimated by the Kalman filter are used to correct the velocity, position, attitude, and inertial sensor measurements of the inertial navigation system, which means that the reference trajectory is corrected and updated periodically, and thus, the state filtering loop will affect the gain calculation loop through the calculation of $\Phi_{k/k-1}$. However, the changes in position induced by typical spoofing attacks do not significantly influence the $\Phi_{k/k-1}$ calculation. A simple and intuitional numerical demonstration is given in Appendix C, in which an assumption of a 5 km position error is introduced to each of the three dimensions. The maximum relative change of each element of $\Phi_{k/k-1}$ is less than 2.5%. This is because a position change of several kilometers is only a small perturbation compared to the earth radius for the $\Phi_{k/k-1}$ calculation, as shown in Appendix A. Generally speaking, the gain calculation loop is weakly correlated with the state filtering loop.

As explained above, the spoofing profile introduced to the state filtering loop will have little impact on the $\Phi_{k/k-1}$ calculation, so $P_{k}$ will basically remain unchanged under the assumption that the other terms ($Q_{k}$, $R_{k}$, $H_{k}$) and the filter initialization are exactly the same under spoofed and authentic conditions. This can be verified from the simulations in Section 5. In other words, $P_{k}$ can no longer reflect the filter’s performance under spoofing attacks. The basic premise of using $P_{k}$ for performance evaluation is proper modeling of both system dynamics and observations. Under spoofing attacks, the measurement model is considered to be incorrect, because the spoofing profile is not modeled and the constant $R_{k}$ cannot reflect the error of the spoofed GNSS measurements. The measurement model used under authentic conditions is no longer valid when the spoofing attacks are introduced. This is the main reason why $P_{k}$ is unreliable and cannot reflect the filter’s performance under spoofing attacks. Therefore, $P_{k}$ should be used cautiously to evaluate the navigation performance, taking spoofing attacks into account.

In civil aviation, the commonly used integrity monitoring algorithms, Honeywell Inertial GPS Hybrid (HIGH) and Autonomous Integrity Monitored Extrapolation (AIME), calculate the horizontal protection level based on $P_{k}$ [27]. Under GNSS spoofing attacks, if the spoofed signals are not detected and removed in time, the horizontal protection level derived from $P_{k}$ is unreliable, which may cause potential integrity risk. Here, we recommend that the integrity algorithm designers reconsider and evaluate the integrity monitoring methods that rely on or relate to $P_{k}$, with spoofing attacks taken into consideration.

### 4.2. Kalman Filter Innovation

The Kalman filter innovations are often used to construct the fault detection statistics for INS/GNSS integrated navigation systems. It is necessary to analyze the effects of spoofing attacks on the Kalman filter innovations to gain a better understanding about spoofing detection methods that are based on them. The discrete system dynamics and measurement model can be written as [22].

(15) $$x_{k}=\Phi_{k/k-1}x_{k-1}+W_{k-1},$$

(16) $$z_{k}=H_{k}x_{k}+V_{k}.$$

The Kalman filter time and measurement update process are implemented in accordance with Figure 2. The Kalman filter innovation, $\upsilon_{k}$, is defined as [28]

(17) $$\upsilon_{k}=z_{k}-H_{k}{\hat{x}}_{k/k-1}.$$

Under normal conditions, when the filter approaches a steady state, the innovation has zero expectation and known covariance [28], that is

(18) $$E\left(\upsilon_{k}\right)=0,$$

(19) $$P_{\upsilon_{k}}=H_{k}P_{k/k-1}H_{k}^{T}+R_{k}.$$

The test statistic of a Chi-squared test for fault detection in the INS/GNSS integrated navigation system at epoch *k* has the simplest form as [22]

(20) $$\beta_{k}=\upsilon_{k}^{T}P_{\upsilon_{k}}^{-1}\upsilon_{k}.$$

If there is no failure, the test statistic obeys a central Chi-squared distribution with *m* degrees, where *m* is the dimension of the measurement vector. The detection threshold is determined given a constant false alarm rate based on the Chi-squared distribution. The Kalman filter innovations can be used to construct a variety of test statistics, and Equation (20) gives the simplest and most commonly used one. The mean of the innovations can be tested and the innovations within a time window can be accumulated or averaged to build different forms of Chi-squared tests [19,23,29].

GNSS spoofing detection is a complex fault detection problem, in which the spoofing attacks can be modeled as drifts or abrupt changes in the sensor measurements. Change in the expectation of the innovation sequence under a drift fault has been investigated in the pressure sensor drift detection problem of a nuclear power plant [30]. Here, we generally follow the derivation given in [30] but extend the fault profile from a simple ramp manner with a fixed rate to a general spoofing attack vector to clearly show the statistical property of the innovation sequence under spoofing attacks. Assume that the spoofing vector, $S_{n}$ (*n* = 0, 1, 2, 3…), is introduced from $t_{\mathrm{Init}}$. For simplicity, we use the new subscript, *n*, to represent the time epoch in a spoofing attack. Now the measurement model becomes

(21) $$z_{n,s}=H_{n}x_{n}+V_{n}+S_{n}.$$

The Kalman filter innovation under spoofing attacks turns to

(22) $$\begin{array}{ll} \upsilon_{n,s} & =z_{n,s}-H_{n}{\hat{x}}_{n/n-1}\\ & =H_{n}x_{n}+V_{n}+S_{n}-H_{n}{\hat{x}}_{n/n-1}\\ & =H_{n}\left(x_{n}-{\hat{x}}_{n/n-1}\right)+V_{n}+S_{n} \end{array}.$$

Define the one-step prediction error at time *n* as

(23) $${\overset{⌣}{x}}_{n}=x_{n}-{\hat{x}}_{n/n-1}.$$

Then, the expectation of the innovation under spoofing attacks is

(24) $$E\left(\upsilon_{n,s}\right)=H_{n}E\left({\overset{⌣}{x}}_{n}\right)+S_{n}.$$

The measurement update equation can be rewritten as

(25) $${\hat{x}}_{n}={\hat{x}}_{n/n-1}+K_{n}H_{n}\left(x_{n}-{\hat{x}}_{n/n-1}\right)+K_{n}\left(V_{n}+S_{n}\right).$$

Multiply both sides of Equation (25) by the state transition matrix $\Phi_{n+1/n}$ to get

(26) $${\hat{x}}_{n+1/n}=\Phi_{n+1/n}\left(I-K_{n}H_{n}\right){\hat{x}}_{n/n-1}+\Phi_{n+1/n}K_{n}H_{n}x_{n}+\Phi_{n+1/n}K_{n}\left(V_{n}+S_{n}\right).$$

Define

(27) $$\Psi_{n}=\Phi_{n+1/n}\left(I-K_{n}H_{n}\right).$$

Then,

(28) $${\hat{x}}_{n+1/n}=\Psi_{n}{\hat{x}}_{n/n-1}+\Phi_{n+1/n}K_{n}H_{n}x_{n}+\Phi_{n+1/n}K_{n}\left(V_{n}+S_{n}\right).$$

Based on the system dynamic model,

(29) $$x_{n+1}=\Phi_{n+1/n}x_{n}+W_{n}.$$

We obtain the one-step prediction error at time *n* + 1 by subtracting Equation (28) from (29):

(30) $${\overset{⌣}{x}}_{n+1}=\Psi_{n}{\overset{⌣}{x}}_{n}-\Phi_{n+1/n}K_{n}\left(V_{n}+S_{n}\right)+W_{n}.$$

Then, the expectation of ${\overset{⌣}{x}}_{n+1}$ is

(31) $$E\left({\overset{⌣}{x}}_{n+1}\right)=\Psi_{n}E\left({\overset{⌣}{x}}_{n}\right)-\Phi_{n+1/n}K_{n}S_{n}.$$

Given a fault free initial condition, $E\left({\overset{⌣}{x}}_{0}\right)=0$, the expectation of ${\overset{⌣}{x}}_{n}$ can be obtained from

(32) $$E\left({\overset{⌣}{x}}_{n}\right)=-\sum_{i=0}^{n-1}\Lambda_{n-1,i}\Phi_{i+1/i}K_{i}S_{i},$$

where

(33) $$\Lambda_{n,i}=\{\begin{array}{cc} \prod_{j=0}^{n-1-i}\Psi_{n-j}, & n>i\\ I, & n=i \end{array}.$$

Finally, we obtain the analytical expectation of the Kalman filter innovation under spoofing attacks:

(34) $$E\left(\upsilon_{n,s}\right)=S_{n}-H_{n}\sum_{i=0}^{n-1}\Lambda_{n-1,i}\Phi_{i+1/i}K_{i}S_{i}.$$

From Equation (34), it is obvious that the spoofing profile at the current epoch has a direct effect on the expectation of the current innovation, while the spoofing profile history has indirect, but accumulated, impacts. If the spoofing profile of each epoch is known, Equation (34) can be solved recursively. As the expectation of the innovation changes under spoofing attacks, carrying out a statistical test directly on the mean of the innovation is a simple and straightforward spoofing detection method. For the error covariance of the innovation, if the spoofing vector does not include additional measurement noise, the error covariance will remain the same as that defined in Equation (19), that is

(35) $$P_{\upsilon_{n,s}}=H_{n}P_{n/n-1}H_{n}^{T}+R_{n}.$$

Meanwhile, the test statistic in Equation (20) does not obey a central Chi-squared distribution anymore, but changes to a non-central Chi-squared distribution with the non-centrality parameter calculated with [19,21]

(36) $$q_{n}=E{\left(\upsilon_{n,s}\right)}^{T}P_{\upsilon_{n,s}}^{-1}E\left(\upsilon_{n,s}\right).$$

The change in the statistical property of the innovations under spoofing attacks leads to a series of innovation-based spoofing detection methods. Furthermore, Tanil [19,21] established a novel, worst-case spoofing profile aimed at maximizing the position error without being detected, based on a similar expression of Equation (34) with slightly different derivations. The worst-case spoofing profile was used instead of the typical ramp and step fault profile that is commonly seen in the GNSS community to evaluate the spoofing detection performance. A linearized Kalman filter implementation was used in [19,21], in which a predefined and known nominal trajectory greatly simplified the spoofing detection and evaluation problem. However, the application of the linearized Kalman filter for INS/GNSS integration is limited to very certain type of applications, like the precision approach, orbiting satellites or interplanetary travel. For the more commonly used extended Kalman filter integration without a predefined reference trajectory, with linearization carried out along the real-time estimation, the spoofing profile is tightly coupled with the Kalman filter real-time estimation, so the worst-case spoofing profile derivation is not applicable. For the general INS/GNSS integration navigation system, typical ramp and step fault profiles (corresponding to synchronized and unsynchronized spoofing attacks) are used in this paper for the simulation analysis. The existence and derivation of a more advanced or, so-called, worst-case spoofing profile that maximizes the integrity risk still needs further effort. The analytical expression derived here could serve as a basis for further investigations.

### 4.3. Inertial Sensor Bias Estimation

In addition to introducing errors in the position and velocity solutions, the spoofing attacks also affect the estimation of inertial sensor biases. Following the derivation in Section 4.2, as we have already obtained the expectation of the one-step prediction error, ${\overset{⌣}{x}}_{n}$, and the expectation of the innovation, $\upsilon_{n,s}$, we can further obtain the expectation of the inertial sensor bias estimation error under spoofing attacks using the following derivation.

First, define the state estimation error at time *n* as

(37) $${\tilde{x}}_{n}=x_{n}-{\hat{x}}_{n}.$$

The relationship of the state estimation error, ${\tilde{x}}_{n}$, and the one-step prediction error, ${\overset{⌣}{x}}_{n}$, can be obtained by combining Equation (37) and Equation (23). Then, we get

(38) $${\tilde{x}}_{n}={\overset{⌣}{x}}_{n}-\left({\hat{x}}_{n}-{\hat{x}}_{n/n-1}\right).$$

The state estimation is given by

(39) $${\hat{x}}_{n}={\hat{x}}_{n/n-1}+K_{n}\left(z_{n,s}-H_{n}{\hat{x}}_{n/n-1}\right)={\hat{x}}_{n/n-1}+K_{n}\upsilon_{n,s}.$$

Substituting Equation (39) into Equation (38), we get

(40) $${\tilde{x}}_{n}={\overset{⌣}{x}}_{n}-K_{n}\upsilon_{n,s}.$$

As the expectations of ${\overset{⌣}{x}}_{n}$ and $\upsilon_{n,s}$ have already been given in Equations (32) and (34), respectively, the expectation of the state estimation error can be obtained with

(41) $$\begin{array}{ll} E\left({\tilde{x}}_{n}\right) & =E\left({\overset{⌣}{x}}_{n}\right)-K_{n}E\left(\upsilon_{n,s}\right)\\ & =\left(K_{n}H_{n}-I\right)\sum_{i=0}^{n-1}\Lambda_{n-1,i}\Phi_{i+1/i}K_{i}S_{i}-K_{n}S_{n} \end{array},$$

where the term, $\Lambda_{n,i}$, has been defined in Equation (33). Recall that the definition of the state vector is $x\left(t\right)={\left[\begin{array}{ccccc} \phi^{T} & {\left(\delta v^{n}\right)}^{T} & {\left(\delta v\right)}^{T} & {\left(\varepsilon^{b}\right)}^{T} & {\left(\nabla^{b}\right)}^{T} \end{array}\right]}^{T}$; the last six elements of $E\left({\tilde{x}}_{n}\right)$ give the expectation of the error of inertial sensor bias estimation. As with the explanation given under Equation (34), the inertial sensor bias estimation is affected by the spoofing profile in an accumulated way. If the spoofing profile of each epoch is exactly known, Equation (41) can be solved. However, as we pointed out at the end of Section 4.2, when the extended Kalman filter is used, the spoofing vector will be tightly coupled with the real-time state estimation, which makes it difficult to build a quantitative relationship between a given spoofing profile and its impacts on the inertial sensor bias estimation without a full simulation analysis. We use a simple analytical demonstration below to give a qualitative analysis here. A simulation is given in Section 5 for more intuitional and quantitative demonstration.

As a common way to demonstrate and analyze the error propagation, we consider a simple static situation of the north channel for qualitative analysis of spoofing impacts on the inertial sensor bias estimation. The north position, north velocity and east misalignment angle error equation can be simplified with Equations (42)–(44), respectively [31].

(42) $$\delta {\dot{r}}_{N}=\delta v_{N},$$

(43) $$\delta {\dot{v}}_{N}=g\phi_{E}+\nabla_{N},$$

(44) $${\dot{\phi }}_{E}=-\delta v_{N}/R+\varepsilon_{E},$$

where $\delta r_{N}$, $\delta v_{N}$ and $\phi_{E}$ are the north position error, north velocity error and east misalignment angle, respectively. $\nabla_{N}$ and $\varepsilon_{E}$ represent the north accelerometer bias and the east gyro bias, respectively. g and R represent the gravity and the earth radius, respectively. Assuming that the spoofer introduces a north position or velocity error, as shown in Equation (43), both the east misalignment angle and north accelerometer bias will be affected. Furthermore, when the east misalignment angle is influenced, the east gyro bias is affected through Equation (44). Generally speaking, a spoofing attack on the north will introduce estimation errors on the north accelerometer bias, east gyro bias, and east misalignment angle. Note that the above analysis only serves as a simple demonstration. The impacts will be much more complicated due to cross coupling of different errors in dynamic situations.

As the gyro and accelerometer bias estimations are affected by the spoofing attacks, spoofing attacks may be detected by monitoring the bias estimation of the inertial sensors from the Kalman filter. This can be done by directly setting upper and lower bounds on the estimated biases. Based on the authors’ experience, the threshold should be set at least three to five times larger than a nominal value provided by the manufacturer. This method is generally conservative, and the user should take the risk that the inertial sensors suffer from performance degradation for other reasons rather than spoofing attacks.

After successful detection of the spoofing attacks, the integrated navigation filter should discard the GNSS solutions if spoofed signals are not removed in time. The navigation filter will output a pure INS solution instead. Conventionally, the estimated inertial sensor biases are used to compensate the raw gyro and accelerometer measurements in the pure INS solution when GNSS measurements are not available for jamming or blockage. In the spoofing scenario, however, the estimated inertial sensor biases are no longer reliable and should not be used. Meanwhile, the spoofing attacks can also affect the misalignment angle estimation, as illustrated above, which also leads to large, pure INS errors. To mitigate the spoofing impacts, the backtracking mechanism, which records the raw IMU measurements in a time window and starts the pure INS solution from a backward time, is recommended after successful spoofing detection. This mechanism has been applied to the initial alignment [32] and can be transferred to spoofing mitigation with minor modifications.

## 5. Simulation Analysis

**Figure 3:**
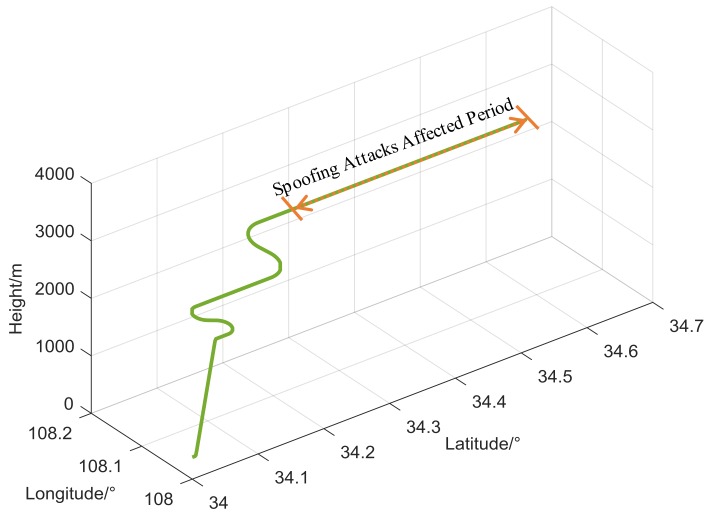
Simulated flight trajectory.

To verify our analyses, we carried out a series of simulations based on the proposed measurement-level trajectory spoofing simulator presented in Section 3.2. We directly fed the spoofing and authentic trajectories to the GNSS measurement-level simulator and switched from authentic to spoofing trajectories during the simulation to introduce the designed spoofing attacks. A GNSS positioning engine was implemented separately into the simulation, with spoofed pseudoranges as inputs and position solutions as outputs for the loosely coupled integration. A flight trajectory lasting 1100 s near Xi’an, China was simulated as the authentic trajectory. The simulated position profiles are given in Figure 3. The parameters of the Kalman filter used in the simulation are listed in Appendix D.

We introduced the spoofing attacks (in the longitude channel in all cases) in the straight-and-level flight period, starting at 800 s; the total period of spoofing was 300 s. For the GNSS simulation, we used the baseline 24-slot GPS constellation at an epoch of 00:00:00 on 1 July 1993 [33], which is also recommended in the RTCA/DO-229 MOPS for INS/GPS system evaluation. The aviation-grade INS and single-frequency GPS receiver were adopted in the simulation with the specifications listed in Table 1. All the following simulations were carried out under the same dynamic scenario as described above.

**Figure 4:**
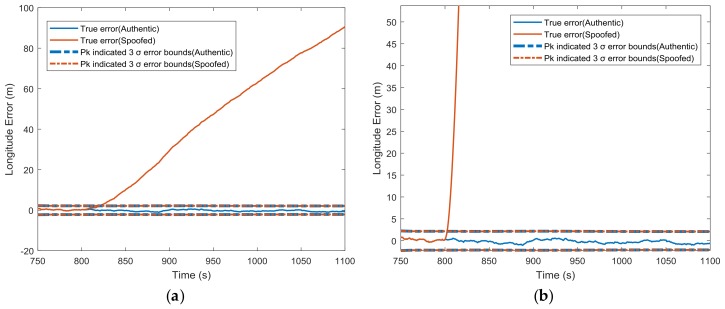
Error covariance matrix ($P_{k}$)-indicated and true position errors under normal conditions and (**a**) 0.3 m/s synchronized spoofing attack; (**b**) 12.4 m/s synchronized spoofing attack.

Figure 4 gives the comparison between the covariance matrix-indicated position errors and true position errors in the longitude channel for both authentic and spoofed scenarios. No matter whether the spoofer introduced a small ramp spoofing profile (0.3 m/s, leads to 90 m longitude error) or a fairly larger one (12.4 m/s, leads to about 2 nautical miles longitude error), the error covariance matrix ($P_{k}$)-indicated error bounds remained the same as the authentic signal, while the actual position errors obviously grew out of the error bounds.

We further compared each diagonal element of $P_{k}$ under spoofed and authentic conditions for all of the estimated states in the scenario shown in Figure 4b at *t* = 1100 s, when about 2 nautical miles longitude error had been introduced. The absolute change in the square root of each diagonal element of $P_{k}$ was defined as

(45) $$\Delta sqrtPk=\left|\sqrt{P_{k,s\left(i,i\right)}}-\sqrt{P_{k,a\left(i,i\right)}}\right|,$$

where the subscripts, *s* and *a*, represent the spoofed and authentic conditions, respectively. *i* = 1, 2, 3, …, 15 represents the index of the diagonal element of $P_{k}$. The relative change was defined as

(46) $$\delta sqrtPk=\frac{\Delta sqrtPk}{\sqrt{P_{k,a\left(i,i\right)}}}.$$

Both the absolute change and relative change are given in Table 2, which clearly shows that the changes in $P_{k}$-indicated state estimation errors were nearly negligible. This verifies that spoofing attacks injected into the state filtering loop have little impact on Kalman filter error covariance calculation. Using $P_{k}$ to evaluate the integrated navigation system’s performance and for integrity monitoring is unreliable under spoofing attacks.

**Figure 5:**
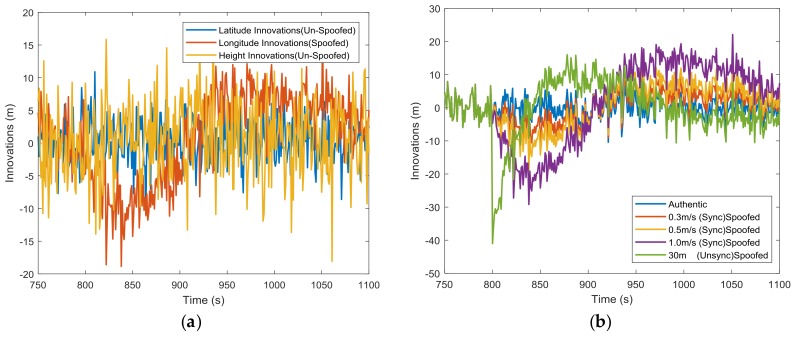
(**a**) Three dimensional Kalman filter innovations; (**b**) longitude channel Kalman filter innovations. (Sync) is short for synchronized and (Unsycn) is short for unsynchronized.

Figure 5a shows the three dimensional innovations under a 0.5 m/s spoofing attack. The abnormal innovations were found only in the longitude channel, because the spoofing attacks introduce only longitudinal errors. Separately, tests on the mean of each component of the innovation vector have the potential to detect and distinguish which component is affected. In Figure 5b, the longitude channel innovations are given for different spoofing profiles, which shows that it may be easier to detect spoofing attacks with larger fault ramps. Also, there is only a limited spoofing detection window [34], as the Kalman filter dynamically tunes itself to track the spoofing profiles, and the means of innovations will approach to zero again in a new steady state. In addition to testing the means of innovations, the other innovation-based tests, like the snapshot Chi-squared test [22,23] or averaged/summed innovation test [19,23,29], can also be used.

**Figure 6:**
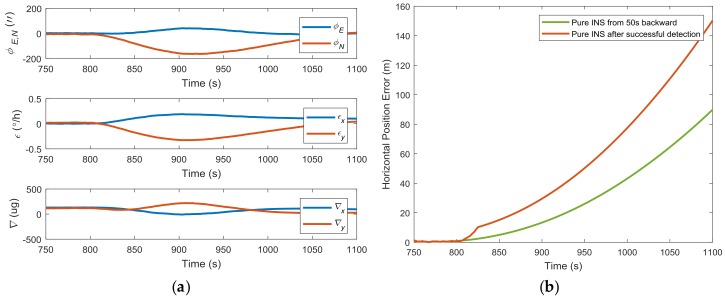
(**a**) Inertial sensor bias estimation and misalignment angle under a 1 m/s synchronized spoofing attack; (**b**) horizontal position accuracy comparison between two pure INS modes.

The impacts of spoofing attacks on the inertial sensor bias and misalignment angle estimation are illustrated in Figure 6a. Taking the gyro bias estimation as an example, the Kalman filter gives an estimation of the *y*-axis gyro bias of over 0.3 °/h under a 1 m/s synchronized spoofing attack, far beyond the 0.01 °/h specification. This gives an obvious sign that abnormal GPS measurements exist. Assuming that the spoofing attack is detected at 825 s, and the GPS measurements in the integrated system are directly disconnected, the horizontal error of pure INS grows quickly to about 150 m, as shown in Figure 6b. When the backtracking mechanism given in [32] (with 50 s backtracking) was used, the unaffected inertial sensor bias and misalignment angle estimation contributed to a better pure INS solution performance with position errors about 90 m. This shows the potential benefits of the backtracking mechanism for spoofing mitigation.

## 6. Conclusions and Future Works

Understanding the behavior of INS/GNSS integrated navigation systems under spoofing attacks is crucial to allow the development of effective spoofing detection and mitigation methods. In this paper, we analyzed the spoofing impacts with a focus on the Kalman filter of the integrated navigation system. Through theoretical analysis and simulations, we came to the conclusion that (1) Kalman filter state error covariance is not affected by spoofing attacks and thus, should be reconsidered for performance evaluation and integrity monitoring; (2) the statistical properties of innovations have changed, which leads to several potential statistical tests for spoofing detection; (3) the estimated inertial sensor biases are no longer reliable under spoofing attacks, which makes bias estimation range check and the backtracking mechanism promising for spoofing detection and mitigation.

What we touched on in this paper is only the tip of the iceberg; this paper opens many research opportunities for the integrated navigation community, taking spoofing attacks into consideration. Simulations in this paper give an intuitional view of the possibility of innovation-based and inertial sensor bias monitoring methods for spoofing detection. Detailed investigations of the recommended spoofing defenses with a comparison and maybe a combination of these defenses deserve further efforts.

## Figures and Tables

**Table 1:** Inertial measurement unit (IMU) and GPS specifications [23].

Sensor	Parameter	Value	Unit
IMU	Gyro bias	0.01	°/h
	Gyro random walk	0.005	${}^{\circ}/\sqrt{h}$
	Accelerometer bias	100	μg
	Accelerometer random walk	20	$\mu g/\sqrt{\mathrm{Hz}}$
GPS	Residual satellite clock and ephemeris errors	0.5	m
	Residual ionosphere error (single-frequency)	4.0	m
	Residual troposphere error	0.2	m
	Tracking noise	0.67	m
	Short-range multipath error	0.94	m

Note: all the specifications are root mean square (RMS) values.

**Table 2:** The absolute and relative change of the square root of each diagonal element of $P_{k}$.

State	Absolute (Relative)	State	Absolute (Relative)	State	Absolute (Relative)
$\phi_{Ε}$	3.43 × 10^−6^° (0.20%)	$\phi_{N}$	4.40 × 10^−6^° (0.26%)	$\phi_{U}$	1.06 × 10^−5^° (0.19%)
$\delta v_{E}^{n}$	1.52 × 10^−6^ m/s (0.01%)	$\delta v_{N}^{n}$	1.96 × 10^−5^ m/s (0.14%)	$\delta v_{U}^{n}$	8.63 × 10^−7^ m/s (0.02%)
δL	3.69 × 10^−4^ m (0.05%)	δλ	1.02 × 10^−5^ m (0.002%)	δH	2.87 × 10^−5^ m (0.004%)
$\varepsilon_{R}^{b}$	1.59 × 10^−5^ °/h (0.11%)	$\varepsilon_{F}^{b}$	2.52 × 10^−5^ °/h (0.18%)	$\varepsilon_{U}^{b}$	7.72 × 10^−6^ °/h (0.03%)
$\nabla_{R}^{b}$	6.33 × 10^−2^ μg (0.29%)	$\nabla_{F}^{b}$	7.17 × 10^−2^ μg (0.35%)	$\nabla_{U}^{b}$	1.14 × 10^−3^ μg (0.07%)

Note: absolute and relative represent the absolute change, ΔsqrtPk, as defined in Equation (45), and relative change,δsqrtPk, as defined in Equation (46), respectively.

## Appendix A

The details of each component of the system matrix, F(t) can be written as

(A1) $$F_{11}=\left(-\left(\omega_{ie}^{n}+\omega_{en}^{n}\right)\times \right),$$

(A2) $$F_{12}==\left[\begin{array}{ccc} 0 & -1/R_{Mh} & 0\\ 1/R_{Nh} & 0 & 0\\ \tan L/R_{Nh} & 0 & 0 \end{array}\right],$$

(A3) $$F_{13}=\left[\begin{array}{ccc} 0 & 0 & v_{N}^{n}/R_{Mh}^{2}\\ -\omega_{ie}\sin L & 0 & -v_{E}^{n}/R_{Nh}^{2}\\ \omega_{ie}\cos L+v_{E}^{n}\sec^{2}L/R_{Nh} & 0 & -v_{E}^{n}\tan L/R_{Nh}^{2} \end{array}\right],$$

(A4) $$F_{21}=\left(\left(C_{b}^{n}f^{b}\right)\times \right),$$

(A5) $$F_{22}=(v^{n}\times )F_{12}-((2\omega_{ie}^{n}+\omega_{en}^{n})\times ),$$

(A6) $$F_{23}=(v^{n}\times )\left[\begin{array}{ccc} 0 & 0 & v_{N}^{n}/R_{Mh}^{2}\\ -2\omega_{ie}\sin L & 0 & -v_{E}^{n}/R_{Nh}^{2}\\ 2\omega_{ie}\cos L+v_{E}^{n}\sec^{2}L/R_{Nh} & 0 & -v_{E}^{n}\tan L/R_{Nh}^{2} \end{array}\right],$$

(A7) $$F_{32}=\left[\begin{array}{ccc} 0 & 1/R_{Mh} & 0\\ \sec L/R_{Nh} & 0 & 0\\ 0 & 0 & 1 \end{array}\right],$$

(A8) $$F_{33}=\left[\begin{array}{ccc} 0 & 0 & -v_{N}^{n}/R_{Mh}^{2}\\ v_{E}^{n}\sec L\tan L/R_{Nh} & 0 & -v_{E}^{n}\sec L/R_{Nh}^{2}\\ 0 & 0 & 0 \end{array}\right],$$

where

(*)× denotes the skew-symmetric matrix of the corresponding vector.

$R_{Mh}$ and $R_{Nh}$ are the meridian and transverse radii of the curvature plus the height.

$v^{n}=\left[\begin{array}{c} v_{E}^{n}\\ v_{N}^{n}\\ v_{U}^{n} \end{array}\right]$ is the navigation frame velocity vector, consisting of east, north, and up components.

L represents the latitude.

$C_{b}^{n}$ is the 3-by-3 body frame to navigation frame rotation matrix, defined as

(A9) $$C_{b}^{n}=\left[\begin{array}{ccc} \cos\gamma \cos\psi +\sin\gamma \sin\psi \sin\theta & \sin\psi \cos\theta & \sin\gamma \cos\psi -\cos\gamma \sin\psi \sin\theta \\ -\cos\gamma \sin\psi +\sin\gamma \cos\psi \sin\theta & \cos\psi \cos\theta & -\sin\gamma \sin\psi -\cos\gamma \cos\psi \sin\\ -\sin\gamma \cos\theta & \sin\theta & \cos\gamma \cos\theta \end{array}\theta \right],$$

where θ,γ,ψ represent the pitch, roll and yaw angle, respectively.

$f^{b}=\left[\begin{array}{c} f_{R}^{b}\\ f_{F}^{b}\\ f_{U}^{b} \end{array}\right]$ is the body frame specific force vector, consisting of right, forward and up components.

$\omega_{ie}$ is the constant earth rotation rate (=7.292115 × 10^−5^ rad/s).

$\omega_{ie}^{n}$ is the earth rotation vector resolved into the local navigation frame, defined as

(A10) $$\omega_{ie}^{n}=\left[\begin{array}{c} 0\\ \omega_{ie}\cos L\\ \omega_{ie}\sin L \end{array}\right].$$

$\omega_{en}^{n}$ is the rotation vector of the navigation frame with respect to the earth-fixed-earth-centered frame represented in the local navigation frame, defined as

(A11) $$\omega_{en}^{n}=\left[\begin{array}{c} -v_{N}^{n}/R_{Mh}\\ v_{E}^{n}/R_{Nh}\\ v_{E}^{n}\tan L/R_{Nh} \end{array}\right].$$

## Appendix B

**Table A1:** A comparison between unsynchronized and synchronized spoofing attacks.

	Parameters	Unsynchronized Spoofing	Synchronized Spoofing
**Signal**	Signal power	$P_{s}>P_{a}$	$P_{s}<<P_{a}\to P_{s}>P_{a}$
	Signal delay	Arbitrarily determined for simulator attacks; generally, $\tau_{s}>\tau_{a}$ for meaconing attacks	$\tau_{s}\approx \tau_{a}$ in the transition process, then determined arbitrarily by the spoofer
	Doppler	Consistent with the spoofer’s code phase variations	$f_{d,s}\approx f_{d,a}$ in the transition process, then varies consistently with the code phase
	Visible satellites	Generally, $N_{s}$ equals $N_{a}$	
	Data sequence	Same structure as $D_{a}$ the content can be changed	Same as $D_{a}$ in the transition process, then the content can be changed
	Code sequence	Strictly the same as the authentic signals	
	Carrier frequency	Strictly the same as the authentic signals	
**Measurement**	Pseudorange	Arbitrarily determined	Matched with $\rho_{a}$ in the transition process, then determined by the spoofer
	Carrier phase	Consistent with code phase, hard to align with the authentic signals	
**PVT**	Position	Arbitrarily determined	Matched with the authentic condition in the transition process, then determined arbitrarily by the spoofer
	Velocity	Consistent with position	
	Time	Arbitrarily determined for simulator attacks; generally earlier than true time for meaconing attacks	
**Implementation**	Platform	Repeater Simulator	Advanced repeater Receiver/spoofer
	Requirements	Jam-and-spoof	Know the real-time position of the target receiver

## Appendix C

To give a simple and intuitional demonstration of the effect of introduced position error on the system state transition matrix Φ calculation, we take a snapshot of the simulation scenario presented in Section 5. Under the authentic condition, at *t* = 1100 s, the snapshot system state is given as

- Position: L = 34.6887°, λ = 108.1915°, H = 2282.8941 m
- Velocity: $v_{E}^{n}$ = −0.0058 m/s, $v_{N}^{n}$ = 149.9911 m/s, $v_{U}^{n}$ = 0.0081 m/s
- Attitude: θ = −0.0018°, γ = 0.0017°, ψ = 359.9941°
- Specific force: $f_{R}^{b}$ = −0.0128 m/s^2^, $f_{F}^{b}$ = −0.0016 m/s^2^, $f_{U}^{b}$ = 9.7900 m/s^2^
- Meridian radius of curvature plus height: $R_{Mh}$ = 6,358,383 m
- Transverse radius of curvature plus height: $R_{Nh}$ = 6,387,345 m

Assuming that 5 km position errors (denoted by δL, δλ and δH) are directly added to the reference trajectory for all the three dimensions, elements of the state transition matrix, $\Phi_{i,j}$ (element in the *i*th row and *j*th column), are calculated before and after the introduction of position errors. Let $\Phi_{i,j}{}_{\left(a\right)}$ and $\Phi_{i,j}{}_{\left(s\right)}$ represent the element under authentic and spoofed conditions, respectively. The elements of δΦ, representing the relative change of Φ, are defined and calculated as

(A12) $$\delta \Phi_{i,j}=\left|\frac{\Phi_{i,j\left(s\right)}-\Phi_{i,j\left(a\right)}}{\Phi_{i,j\left(a\right)}}\right|,$$

where $\delta \Phi_{i,j}$ is the element in the *i*th row and *j*th column of δΦ. The first order solution, $I+F\left(t\right)T_{s}$, is used to compute the transition matrix, where ***F***(*t*) is the system matrix, as defined in Equation (3) and Appendix A, and $T_{s}$ is the propagation interval which equals 0.01 s in this demonstration. Here, take the calculation of $\Phi_{8,4}$ as an example (corresponding to the term of $\sec L/R_{Nh}$ in Equation (A7)). The relative change of $\Phi_{8,4}$ is calculated as

(A13) $$\delta \Phi_{8,4}=\left|\frac{\Phi_{8,4\left(s\right)}-\Phi_{8,4\left(a\right)}}{\Phi_{8,4\left(a\right)}}\right|=\left|\frac{\left(0+\sec(L+\delta L)/\left(R_{Nh}+\delta H\right)\cdot T_{s}\right)-\left(0+\sec L/R_{Nh}\cdot T_{s}\right)}{0+\sec L/R_{Nh}\cdot T_{s}}\right|.$$

Replacing all the terms with the known numerical values, we get

(A14) $$\delta \Phi_{8,4}=\left|\frac{\left(0+\sec(0.6054+5000/6378137)/\left(6387345+5000\right)\cdot 0.01\right)-\left(0+\sec\left(0.6054\right)/6387345\cdot 0.01\right)}{0+\sec\left(0.6054\right)/6387345\cdot 0.01}\right|\approx 0.02\%,$$

which shows that a very small amount of change has been introduced. All the elements of δΦ are calculated in the same way. Finally, we get the relative change of Φ in percentage form as

(A15) $$\delta \Phi_{i=1:8,j=1:15}=\left[\begin{array}{ccccccccccccccc} -- & 0.01 & 0.05 & -- & 0.08 & -- & -- & -- & 0.16 & -- & 0.09 & 0.57 & -- & -- & --\\ 0.11 & -- & 0.08 & 0.08 & -- & -- & 0.11 & -- & 0.15 & 0.09 & -- & 0.14 & -- & -- & --\\ 0.05 & 0.08 & -- & 0.09 & -- & -- & 0.05 & -- & 0.01 & 0.57 & 0.14 & -- & -- & -- & --\\ -- & -- & 0.03 & -- & 0.11 & 0.05 & 0.05 & -- & 0.01 & -- & -- & -- & -- & 0.09 & 0.57\\ -- & -- & 0.01 & 0.01 & -- & 0.08 & 0.05 & -- & 2.19^{**} & -- & -- & -- & 0.09 & -- & 0.14\\ 0.03 & 0.01 & -- & 0.05 & 0.08 & -- & 0.12 & -- & 0.16 & -- & -- & -- & 0.57 & 0.14 & --\\ -- & -- & -- & -- & 0.08 & -- & -- & -- & 0.16 & -- & -- & -- & -- & -- & --\\ -- & -- & -- & 0.02^{*} & -- & -- & 0.15 & -- & 0.10 & -- & -- & -- & -- & -- & -- \end{array}\right]\%.$$

Note that only part of δΦ is given; all the omitted elements and the elements denoted by “--” are not influenced by introduced position errors (elements with relative change <1 × 10^−9^ are also denoted by “--”) .The superscripts, * and **, are used to indicate $\delta \Phi_{8,4}$ for demonstration in Equation (A14) and $\delta \Phi_{5,9}$ for the maximum relative change, respectively.

## Appendix D

The parameters of the Kalman filter used in the simulation are listed here for reference:

$$P_{o}=\mathrm{diag}{\left(\left[20\prime \prime ;20\prime \prime ;180\prime \prime ;\;0.1\;m/s;\;0.1\;m/s;\;0.1\;m/s;\;3.7\;m;\;3.7\;m;6m;0.01^{o}/h;0.01^{o}/h;0.01^{o}/h;100\;\mu g;100\;\mu g;100\;\mu g\right]\times 3\right)}^{2},$$

$$Q_{k}=\mathrm{diag}{\left(\left[0.005^{o}/\sqrt{h};0.005^{o}/\sqrt{h};0.005^{o}/\sqrt{h};20\;\mu g/\sqrt{\mathrm{Hz}};20\;\mu g/\sqrt{\mathrm{Hz}};20\;\mu g/\sqrt{\mathrm{Hz}};1\;m/\sqrt{h};1\;m/\sqrt{h};1m/\sqrt{h};\mathrm{zeros}(6,1)\right]\right)}^{2}\times Ts,$$

$$R_{k}=\mathrm{diag}{\left(\left[3.7m;3.7m;6m\right]\right)}^{2},$$

$$x_{0}=\mathrm{zeros}(15,1).$$

Note that *Ts* = 0.01 s is the propagation interval in the simulation. The parameters are set according to IMU and GPS specifications, as listed in Table 1. The units given above are just for easy interpretation. They should be changed to SI in practical implementations.
